# Investigating the Phytochemistry and Underlying Glycemic Control Mechanisms of *Litchi chinensis* Sonn. (Litchi) Peel Ethyl Acetate Extract in a Fructose/Streptozotocin Diabetic Model of Rats

**DOI:** 10.3390/nu16213644

**Published:** 2024-10-26

**Authors:** Gloria O. Izu, Samson S. Mashele, Chika I. Chukwuma

**Affiliations:** 1 Department of Health Sciences, Faculty of Health and Environmental Sciences, Central University of Technology, Bloemfontein 9301, Free State, South Africa; 221039621@stud.cut.ac.za; 2 Centre for Quality of Health and Living (CQHL), Faculty of Health and Environmental Sciences, Central University of Technology, Private Bag X20539, Bloemfontein 9300, Free State, South Africa; smashele@cut.ac.za

**Keywords:** antioxidant, ethyl acetate extract, flavonoids, fructose/streptozotocin diabetic rats, glycemic control, litchi fruit peel

## Abstract

The glycemic control potential and flavonoid profile of litchi have been documented for its hydroalcoholic extracts, while there is scarce information regarding its ethyl acetate extract. This study investigated the flavonoid profile, as well as the ameliorative potential and possible underlying mechanisms of litchi peel ethyl acetate extract on type 2 diabetes-related pathologies in a fructose/streptozotocin (STZ) model of diabetic rats. Sprague Dawley rats were induced with diabetes by administering 10% fructose for 2 weeks and a single i.p. injection of low-dose (40 mg/kg bw) STZ. Thereafter, the animals were orally administered with a low-dose (150 mg/kg bw) and high-dose (300 mg/kg bw) of the peel extract (LDPE and HDPE, respectively) and metformin (200 mg/kg bw). Compared to untreated diabetic rats (AUC = 1004 mg.h/dL), the HDPE significantly (*p* < 0.05) improved glucose tolerance (AUC = 847 mg.h/dL), which was statistically comparable (*p* ˃ 0.05) to the effect of metformin (AUC = 903 mg.h/dL). Serum insulin and pancreatic histology data showed that the STZ-induced pancreatic damage and insulin depletion was improved by the HDPE, which could be linked to the observed ameliorative effect of the extract on pancreatic lipid peroxidation and SOD and catalase activity. The extract further improved liver and muscle glycogen storage, as well as muscle hexokinase activity and Akt phosphorylation, suggesting that the extract exerts glycemic control by enhancing glycogen storage and modulating insulin-mediated signaling of glucose uptake and utilization. LC-MS data and documented reports suggest that flavonoids, such as epicatechin, cinnamtannin B2, procyanidin B5, and proanthocyanidin A2, are the possible influencing compounds. The ethyl acetate extract of litchi peel could be a source of bioactive flavonoids that can potentiate glycemic control in diabetes and mitigate oxidative stress-related pathologies.

## 1. Introduction

According to the International Diabetes Federation [1], the global prevalence of adults suffering from diabetes will increase from 537 million in 2021 to 783 million in 2045 [1], suggesting that the negative impact of diabetes on global health and socioeconomic landscapes could worsen if precautionary measures are not implemented. Diabetes is characterized by detrimental metabolic imbalances caused by hormonal insufficiency or dysfunction, which results in persistent hyperglycemia [2]. Type 2 diabetes (T2D) is the most prevalent type of diabetes, possibly due to its association with lifestyle-related factors, such as physical inactivity, dieting, and detrimental weight gain [3]. These factors lead to insulin resistance, which is a key pathophysiological defect of T2D [3,4]. During insulin resistance, the homeostatic regulatory action of insulin on circulating glucose is impaired due to dysfunctional signaling in the cells of peripheral tissues [4]. Cellular glucose uptake, nutrient metabolism, and utilization are impaired, which leads to reduced glycemic control and eventually persistent hyperglycemia, especially if the function of the pancreatic β-cells has been concomitantly compromised [4].

Persistent hyperglycemia has been linked to the development of microvascular and macrovascular complications of diabetes and oxidative stress has been documented as a key underlying mediator [5]. Oxidative stress is caused by an imbalance between the physiological production of deleterious prooxidants or reactive oxygen species (ROS) and the antioxidant mechanisms to mop or quench the oxidant action of the prooxidants [6]. During diabetes, hyperglycemia facilitates the production of ROS, which can oxidatively damage cellular macromolecules and organelles, while signaling associated inflammatory responses [5]. High blood glucose can cause glycation to key functional macromolecules, leading to the production of advanced end products (AGEs), which are biological indicators of degenerative diseases, including diabetic complications [5]. Elevated lipid peroxidation in diabetes has also been documented, which can compromise cellular integrity due to damage to the lipid bilayer of cell membranes [5]. Also, deleterious radical byproducts of lipid peroxidation can increase oxidative stress and cause oxidative damage to macromolecules [5].

While there are many available commercial antidiabetic drugs, medicinal plants, including fruits and vegetables are increasingly been used as complementary and/or alternative therapies. Dietary antioxidant phenolic compounds present in fruits and vegetables offer a broad spectrum of pharmacological activities that could mitigate the development of diabetic complications by suppressing oxidative stress and inflammatory responses [7]. *Litchi chinensis* Sonn. (Sapindaceae) is a tropical and subtropical fruit with high commercial value due to its global consumption [8]. The nutritional value and medicinal benefits of the fruit have also been documented [8]. While studies have mentioned the antioxidant, anti-hyperglycemic, anti-hypertensive, anti-obesogenic, anti-dyslipidaemic, and hepatoprotective potentials of the fruit [8], documented evidence has shown that the non-edible peel could also be of medicinal relevance [9,10]. Flavonoids, including catechins, procyanidins, proanthocyanidins, quercetin, rutin, and their derivatives have been reported in the peel, which have been linked to some of the reported diabetes-related and antioxidant pharmacological properties of the peel [9,10,11]. It has been shown that the fruit peel has a strong α-glucosidase inhibitory potential [12], indicating it could potentiate glycemic control by reducing postprandial blood glucose increase [13]. In high-fat-diet-fed rodents, the peel powder and its procyanidin-rich fraction were shown to improve the profiles of blood lipids and glucose, thus ameliorating diabetes-related metabolic alterations [14,15]. In *db/db* mice, litchi peel ethanol extract treatment has been shown to ameliorate type 2 diabetic pathologies, including elevated blood glucose, hepatic gluconeogenesis, oxidative stress, and associated inflammatory responses [16].

A review of the pharmacology potential of litchi fruit seed and peel [9] suggests that diabetes-related pharmacological investigations have mostly been demonstrated in the hydro-alcoholic extracts or isolates with minimal investigations on other solvent extracts, such as the ethyl acetate extract. Ethyl acetate solvent extraction can cause hydrogen bond formation with carboxyl and hydroxyl groups, resulting in the extraction of medium polar to polar phenolics and/or flavonoids [17], which could influence potent medicinal properties. In fact, documented qualitative evidence has shown that the ethyl acetate extract of litchi peel contains a relatively similar proportion of phenolic compounds and flavonoids as the hydroalcoholic extract [18]. A previous study has proven that litchi fruit ethyl acetate extract demonstrates potent immunomodulatory potential [19]. In a more recent antidiabetic study, a comparative assessment in alloxan-induced type 1 diabetic Wistar rats suggests that the blood glucose lowering, and antioxidant effect of litchi peel ethyl acetate extract outperformed that of the hydroalcoholic extract [18], suggesting that litchi peel ethyl extract may possess ameliorative potential against T2D-associated pathologies. However, this speculation, as well as the possible mechanisms, remain elusive and warrants investigation. Therefore, this study investigated the ameliorative potential and possible underlying mechanisms of litchi peel ethyl acetate extract on T2D-related pathologies in a fructose/low-dose streptozotocin (STZ) model of diabetic rats.

## 2. Materials and Methods

### 2.1. Plant Material and Extraction

Litchi fruit (McLean’s Red cultivar) was purchased from a local fruit and vegetable store in Bloemfontein, South Africa. Clean water was used to wash the fruits, and the peel of the fruits was collected. An oven set at 30 °C was used to dry the peel of the fruits (Model No. 072160, Prolab instrument, Sep Sci., Johannesburg, South Africa). The dried peel was ground. About 300 g of the ground peel was defatted with 1.5 L of hexane. In order to increase polarity, sequentially extraction was conducted on the defatted peel using 1.5 L of dichloromethane (DCM) and ethyl acetate (EtAc) solvents. The extraction was conducted for 48 h at room temperature on an orbital shaker [OrbiShake, Model 262, Labotec 106 (Pty) Ltd., Johannesburg, South Africa] set at 125 rpm. The concentrated ethyl acetate extract was recovered using a Buchi 108 Rotavapor^®^ R-300 [Labotec (Pty) Ltd., Johannesburg, South Africa] and dried under a fume hood. The yield of the extract was determined, before storing it in clean screw-cap vials at −20 °C.

### 2.2. LC-MS Analysis of the Ethyl Acetate Extract of Litchi Peel

To perform the LC-MS protocol, the following were used: A Waters Synapt G2 (Waters Corporation, Milford, MA, USA), an ESI probe, and an ESI Pos, at a 15 V cone voltage. The operation of liquid chromatography was conducted using an Acquity binary solvent manager. The chromatographic column used was a HSS T3 (Waters Corporation, Milford, MA, USA). It had a dimension of 2.1 × 150 mm and a particle size of 1.8 µm. Two solvent systems (A and B) were used as the mobile phase. Solvent system A was water containing 0.1% methanoic acid, while solvent system B was acetonitrile containing 0.1% methanoic acid. A 0.25 mL/min flow rate was applied for the gradient chromatographic separation at 30 °C. The gradient condition followed the following sequence: 100% (A):0% (B), 10 min; 72% (A):28% (B), 21 min; 60% (A):40% (B), 50 s; 0% (A):100% (B), 2 min. The extract was dissolved in a mixture of acetonitrile and water (1:1 v/v), passed through a 0.22 µm filter, and injected at a volume of 20 μL. The signals of the separated compounds were recorded at 254 nm. Using the accurate masses, the compounds in the fraction were tentatively determined and semi-quantified (mg/L) relative to the epicatechin standard and according to the extracted ions.

### 2.3. Animals

The approval for the use of animals in this study was obtained from the University of Free State Animal Research Ethics Committee (EREC) on 07 February 2023 (Protocol reference number: UFS-AED2022/0028/23). Thirty-five 7 to 8-week-old Sprague-Dawley (SD) rats were procured by the University of the Free State Animal Research Unit, where the animal experiment was conducted. The animals were allowed to acclimatize for 5 days. Thereafter, the animals were assigned into five groups containing seven animals each. The groups were named the normal control (NC), diabetic control (DBC), low-dose peel extract (LDPE), high-dose peel extract (HDPE), and metformin (MET) groups. The animals were housed two to three animals per cage in large-sized cages with the following dimensions: 598 mm (L) × 380 mm (W) × 200 mm (H) with Grid top hatch access of 220 mm (L) × 225 mm (W) × 70 mm (H). The animals were housed in a temperature (20–24 °C; set point at 22 °C) and humidity (40–70%; set point 60%) controlled room with a 12 h light-dark cycle.

### 2.4. Diabetes Induction

A previous study [20] has shown that the combination of 10% fructose and low dose (40 mg/kg bw) STZ could be used to develop a diabetic SD rat model that exhibits two key pathologies of T2D, namely insulin resistance and β-cell damage. This approach was adopted to induce diabetes. First, the rats in the diabetic groups (DBC, LDPE, HDPE, and MET groups) were fed ad libitum with 10% fructose solution in place of drinking water for 2 weeks. After that, the rats were injected with a single intraperitoneal (i.p.) injection of STZ (40 mg/kg bw) dissolved in citrate buffer (pH, 4.4), whereas the rats in the NC group were injected with an equivalent volume of citrate buffer. One week after the STZ injection, the tail-tip blood glucose (mmol/L) of rats was measured using a Contour Plus Blood Glucose Monitoring System (Ascensia Diabetes Care Holdings AG, Basel, Switzerland). A multiplication factor of 18.018 was used to convert the blood glucose measurements from mmol/L to mg/dL. Animals with blood glucose values ranging from 250 to 600 mg/dL were considered diabetic and included in the study. Of the 28 rats induced with diabetes, four rats were extremely diabetic (blood glucose > 600 mg/dL) and thus were excluded from the study. Therefore, each of the diabetic groups was left with six animals each. The number of animals in the NC group was also reduced to six to be consistent with other groups.

### 2.5. Treatment

After diabetes confirmation, the rats in the different groups were administered via oral gavage with the following treatments:

- NC group: The vehicle of the extract or metformin (5% DMSO solution);
- DBC group: The vehicle of the extract or metformin (5% DMSO solution);
- LDPE: 150 mg/kg bw of extract dissolved in 5% DMSO solution;
- HDPE: 300 mg/kg bw of extract dissolved in 5% DMSO solution;
- MET: 200 mg/kg bw of Metformin hydrochloride (Merck, Johannesburg South Africa) dissolved in dissolved in 5% DMSO solution.

The animals were treated for 20 days during a 4-week intervention period. This implies that the animals were treated during the first five days of each week of the intervention period.

### 2.6. Measurement of Fluid Intake, Food Intake, and Non-Fasting Blood Glucose

During the intervention period, the average daily fluid intake and average weekly food intake of the animals were measured. The tail-tip non-fasting blood glucose (NFBG) level of the animals was measured on the 6th day of the 1st, 2nd, and 4th weeks of the intervention period. The NFBG was measured using a glucometer.

### 2.7. Measurement of Oral Glucose Tolerance

The oral glucose tolerance test (OGTT) was performed on the 6th day in the 3rd week of the intervention period. The rats were fasted for 7 h (from 3:00 a.m. to 10:00 a.m.). Thereafter, the tail-tip blood glucose of the animals was measured (0 min) before orally administering 2 g/kg bw of glucose. The tail-tip blood glucose of the animals was then measured for 120 min at 30 min intervals. Thereafter, the animals were allowed access to food. The area under the curve (AUC) for the OGTT was calculated according to a previous report [21]:

$$AUC\;(mg\cdot h/dL)=\frac{BG0+\left(BG30\times 2\right)+\left(BG60\times 3\right)+(BG120\times 2)}{4}$$

where BG0, BG30, BG60, and BG120 are the mean blood glucose (mg/dL) at 0, 30, 60 and 120 min, respectively.

### 2.8. Euthanization and Sampling

After the intervention period, the animals were euthanized using isoflurane. The blood of the animals was collected by cardiac puncture and stored in tubes without an anti-coagulant. Also, the liver, pancreas and muscle tissues of the animals were collected. The collected tissue samples were immediately frozen in liquid and stored at −80 °C. A portion of the pancreatic tissues was fixed in 10% neutral buffered formalin (Merck, Johannesburg, South Africa) for histological assessment. The blood was centrifuged at 3500× *g* for 10 min at 4 °C using a NUVE NF 800R centrifuge (Separations, Johannesburg, South Africa) and the supernatant (serum) was stored at −80 °C.

### 2.9. Measurement of Liver and Muscle Glycogen Content

The phenol–sulfuric acid method [22] was used according to a modified version [23]. About 0.5 g of tissue was disintegrated in a vial by adding 1.5 mL of 30% KOH solution and boiling for 40 min to form a homogenous solution. After cooling, 2 mL of 95% ethanol was used to precipitate the sugars in the homogenized tissues. This was performed at 2–8 °C for 30 min. The supernatant was recovered and removed by centrifuging at 1200× *g* for 30 min at ambient temperature. The residue or sediment was washed twice with 95% ethanol and dissolved in 3 mL of distilled water. From the dissolved residue or glycogen standards (0.005–0.1 mg/200 µL), 200 µL was transferred into glass vials. Then, 1 mL of phenol (5% solution) and 5 mL of H_2_SO_4_ (98% solution) were added, successively. The vials were gently agitated to mix them. The absorbance of aliquots (250 µL) from each vial was measured at 490 nm (SpectraMax iD3 multi-mode microplate reader; Molecular Devices, San Jose, CA, USA). From the glycogen standard curve (y = 0.021x − 0.0165; R^2^ = 0.9984), the glycogen content (mg/g tissue) of the tissues was calculated as follows:

$$Tissue\;glycogen\;content\;\left(mg/g\;tissue\right)=\frac{C\times D}{M}\;$$

where “C” is the extrapolated concentration (mg/200 μL); “D” is the dilution factor = 31, which is the dilution of 200 µL of the dissolved residue in 6.2 mL of assay volume; and “M” = 0.5 g, which is the mass (g) of tissue used in the assay.

### 2.10. Measurement of Serum Insulin

Serum insulin was measured using an enzyme-linked immunosorbent assay (ELISA) method. A Rat/Mouse Insulin ELISA Kit (Merck, Johannesburg, South Africa; product number: EZRMI) was used to perform the assay and the serum insulin concentration was expressed in ng/mL.

### 2.11. Tissue Homogenization and Protein Estimation

The tissues were thawed and homogenized using a method reported previously [23]. About 100 mg of the different tissues (liver, pancreas, and muscle) was homogenized in 1 mL of ice-cold buffer (sodium phosphate buffer containing 0.5% *v*/*v* Triton X-100 and 1 mM EDTA, pH 7.5) using a D1000 handheld homogenizer (Merck, Johannesburg, South Africa). The supernatant of the tissue homogenates was recovered at 10,400× *g* for 10 min at 4 °C. The protein concentration in the supernatant was measured. The protein concentration (mg/mL) was extrapolated from a bovine serum albumin (BSA) standard curve (y = 0.7059x + 0.1982; R^2^ = 0.9992) using the bicinchoninic acid (BCA) method.

### 2.12. Measurement of Muscle Tissue Hexokinase Activity

The activity of hexokinase in the muscle tissue homogenate was measured at a protein concentration of 5 mg/mL according to the protocols described in a Hexokinase Colorimetric Assay Kit (Merck, Johannesburg, South Africa; catalogue number, MAK091). Hexokinase catalyzes the phosphorylation of glucose to glucose-6 phosphate. Glucose-6 phosphate is oxidized by glucose-6 phosphate dehydrogenase, during which the oxidized form of nicotinamide adenine dinucleotide (NAD^+^) is reduced to NADH. This assay estimates hexokinase activity by measuring the NADH-mediated colorimetric reduction of a chromogenic probe over 20 min. NADH production was quantified from an NADH standard curve (y = 0.111x − 0.0147; R^2^ = 0.998) of NADH amounts (2.5, 5, 7.5, 10, and 12.5 nmol/reaction mixture) versus the corresponding absorbances at 450 nm. One unit of hexokinase represents the enzyme activity that will generate 1.0 μmol of NADH from NAD^+^ per minute at pH 8.0 under room temperature. The specific activity (nmol/min/mg protein) was calculated to the protein concentration of the tissue homogenates as follows:

$$Specific\;activity\;(nmol/min/mg\;protein)=\frac{A\times DF}{RT\times SV\times PC}$$

where “A” = amount (nmole) of NADH generated during reaction time; “DF” = dilution factor of sample in reaction volume; “RT” = reaction time (min); “SV” = sample volume (mL); and “PC’ = sample protein concentration (mg/mL).

### 2.13. Measurement of Muscle Tissue Akt Phosphorylation

The pan-Akt and phosphor-Akt levels in the muscle tissue supernatant were estimated by a colorimetric ELISA method using a Phospho-Akt (pSer473)/pan-Akt ELISA Kit (Merck, Johannesburg, South Africa; catalogue number, RAB0012) and presented as the spectrometric optical densities (OD). To estimate the levels of Akt phosphorylation, the ratio between the ODs of phospho-Akt and pan-Akt was computed.

### 2.14. Measurement of Lipid Peroxidation

The protocol reported in a previous study [24] was slightly modified to perform this assay. Lipid peroxidation was estimated in the serum and tissue homogenates (liver and pancreas). In a vial, the tissue homogenate or malondialdehyde (MDA) standards (100 µL), 0.25% thiobarbituric acid solution (500 µL), 20% acetic acid solution (200 µL), and distilled water (200 µL) were successively added and mixed. The MDA standards were 7.5, 15, 22.5, 30, 45, and 60 µM solutions. The vials were placed in a boiling water bath for 50 min and the absorbance of aliquots (250 µL) from each vial was measured at 532 nm. Lipid peroxidation, determined as the concentration of thiobarbituric acid reactive substances (TBARS), was extrapolated from the MDA standard curve (y = 0.0083x + 0.0062; R^2^ = 0.999).

### 2.15. Measurement of Superoxide Dismutase Activity

Superoxide dismutase activity (SOD) was measured in the serum and tissue homogenates (liver and pancreas) using a SOD Assay Kit (product number, 19160; Merck, Johannesburg, South Africa). SOD catalyzes the reduction of superoxide ions. Thus, the activity of SOD in the samples was determined by measuring the inhibitory action on superoxide ion-mediated colorimetric (450 nm) reduction of a water-soluble tetrazolium salt [2-(4-Iodophenyl)-3-(4-nitrophenyl)-5-(2,4-disulfophenyl)-2H-tetrazolium, monosodium salt] as follows:

$$Inhibition\;(\%)=\frac{{OD}_{C}\;-{OD}_{S}}{{OD}_{C}}\;\;\times \;100$$

where “OD_C_” is the optical density for colorimetric (450 nm) reduction by the control (without enzyme), while “OD_S_” is the optical density for colorimetric (450 nm) reduction by the samples.

The specific activity (nmol/min/mg protein) was calculated with respect to the protein concentration of the tissue homogenates as follows:

$$Specific\;activity\;(\%\;inhibition/mg\;protein)=\frac{I\times DF}{SV\times PC}$$

where “I” = percentage inhibition (%) by enzyme on superoxide ion-mediated colorimetric reduction of a water-soluble tetrazolium salt; “DF” = dilution factor of sample in reaction volume; “SV” = sample volume (mL); and “PC’ = sample protein concentration (mg/mL).

### 2.16. Measurement of Catalase Activity

Catalase activity was measured in the serum and tissue homogenates (liver and pancreas) using a Catalase (CAT) Activity Assay Kit (Catalogue number, E-BC-K031-S; Biocom Africa, Johannesburg, South Africa). Catalase catalyzes the decomposition of H_2_O_2_, a reaction that can be chromogenically (405 nm) stopped by ammonium molybdate. The activity of catalase in the samples was estimated by measuring the residual H_2_O_2_ after 1 min of enzyme-substrate reaction. The residual H_2_O_2_ was extrapolated from a H_2_O_2_ standard curve (y = 0.0307x − 0.0002; R^2^ = 0.9998) of H_2_O_2_ amounts (0, 0.5, 1, 2, 3, 4, and 5 µmol) versus the corresponding absorbances at 405 nm. The specific activity (U/mg protein) was calculated with respect to the protein concentration of the tissue homogenates as shown below. One unit of catalase will decompose 1.0 micromole of H_2_O_2_ per minute at pH 7.0 under room temperature at a substrate concentration of 50 mM H_2_O_2_.

$$Specific\;activity\;(U/mg\;protein)=\frac{A\times DF}{RT\times SV\times PC}$$

where “A” = amount (µmole) of H_2_O_2_ that was used up during reaction time; “DF” = dilution factor of sample in reaction volume; “RT” = reaction time (min); “SV” = sample volume (mL); and “PC’ = sample protein concentration (mg/mL).

### 2.17. Histopathological Examination of Pancreatic Tissues

The fixed pancreatic tissues were dehydrated by successive changes in concentrations (50%, 70%, 90%, and 100%, respectively) of ethanol. Ethanol was removed from the tissue by repeated immersions in xylene before being embedding in paraffin according to the standard protocol. The embedded tissues were cut into about 4 µm sections and mounted on slides. Hematoxylin and eosin were used to stain tissue samples to highlight important features of the tissue samples. The DPX mounting media was applied before placing a cover slip. The slides were viewed with a Leica slide scanner.

### 2.18. Data and Statistics Presentation

The data of this study were presented as the average of the replicate data (n = six animals) along with the standard deviation (±). For each parameter, statistical analysis was conducted to compare the data mean of the different groups. This was performed using the IBM SPSS Statistics software (version 29). The one-way ANOVA and Tukey post hoc were used for computing the multiple comparisons of the data averages and statistical significance was set at *p* < 0.05.

## 3. Results and Discussion

The antidiabetic and antioxidant potential of litchi peel has been documented [9,12,14,15,16]. However, study trends show that the hydroalcoholic extracts have mostly been studied, while the ethyl acetate extract, which has been shown to have promising antidiabetic, phenolic, and flavonoid profiles [18], remains under-explored. The present study demonstrates the rich flavonoid profile of litchi peel ethyl acetate extract, as well as its ameliorative effects on T2D-related pathologies in a fructose/STZ model of diabetic rats.

### 3.1. Phytochemical Profile of Litchi Peel Ethyl Acetate Extracts

**Figure 1:**
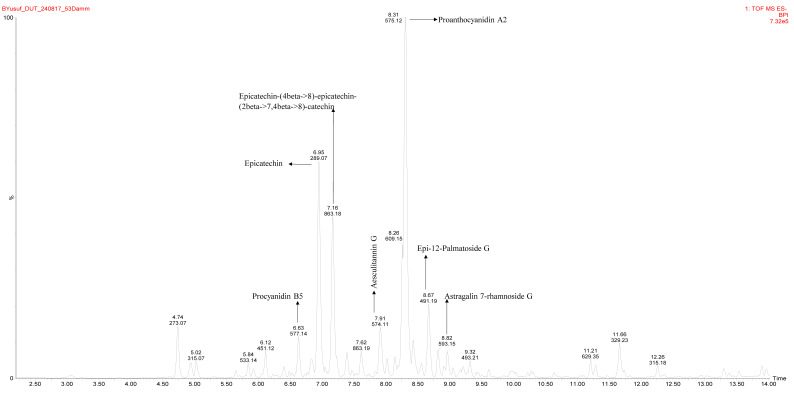
LC-MS chromatogram of litchi peel ethyl acetate extract.

The hydroalcoholic extracts of litchi peel have been extensively studied. Documented evidence has shown that the hydroalcoholic extracts of litchi peel are rich in flavonoids including catechins, proanthocyanidins, and procyanidins, which are believed to be key bioactive flavonoids influencing the antioxidant, antidiabetic, anti-hypertensive, and anti-inflammatory potentials of the hydroalcoholic peel extracts [11,12,16,25]. On the other hand, a previous qualitative assessment [18] has suggested that the ethyl acetate extract of litchi peel may also be as rich in flavonoids as the hydroalcoholic extracts. However, specialized spectrometric profiling is still needed to confirm this. In our study, LC-MS analysis showed the notable presence of flavonoids such as epicatechin, cinnamtannin B2, procyanidin B5, and proanthocyanidin A2 in the ethyl acetate extract of litchi peel (Table 1, Figure 1). The presence of these flavonoids suggests that the ethyl acetate extract may also potentiate antioxidant and antidiabetic effects, which have been investigated and reported in this study.

### 3.2. Glycemic Control and Antioxidant Potential of Litchi Peel Ethyl Acetate

**Figure 2:**
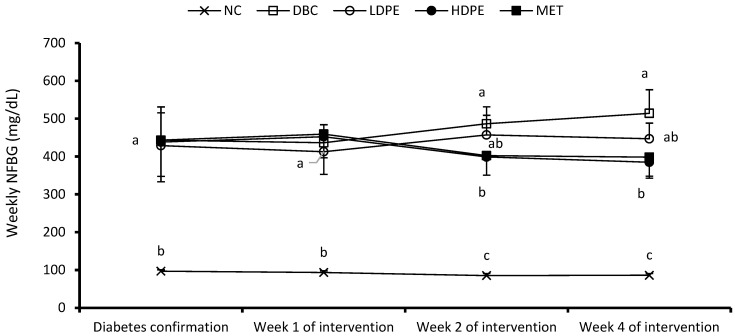
Weekly non-fasting blood glucose (NFBG). “NC”, normal control; “DBC”, diabetic control; “LDPE”, low-dose peel extract; “HDPE”, high-dose peel extract; “MET”, metformin. For each period, a statistical comparison was conducted among the groups. A significant difference (*p* < 0.05) exists between any two groups when there are no similar letters.

Persistent hyperglycemia in a diabetic state causes excessive urinary excretion of glucose, which is responsible for some of the classical symptoms of diabetes, including weight loss, excessive food consumption (polyphagia), and excessive fluid consumption (polydipsia) [26]. In the present study, diabetic rats significantly (*p* < 0.05) lost weight and consumed more food and water compared to their nondiabetic counterparts (Table 2), which is influenced by their higher (*p* < 0.05 blood glucose levels (Figure 2). Treatment with the peel extracts (LDPE and HDPE) and metformin did not significantly improve the diabetic weight loss or fluid intake. The HDPE, however, had an appreciable positive effect on diabetic weight loss and fluid intake particularly in the fourth week of intervention. In fact, although not normalized, the HDPE significantly (*p* < 0.05) reduced diabetic polyphagia (Table 2), suggesting a notable ameliorative effect of the litchi peel ethyl acetate extract, which can be further investigated long-term.

**Figure 3:**
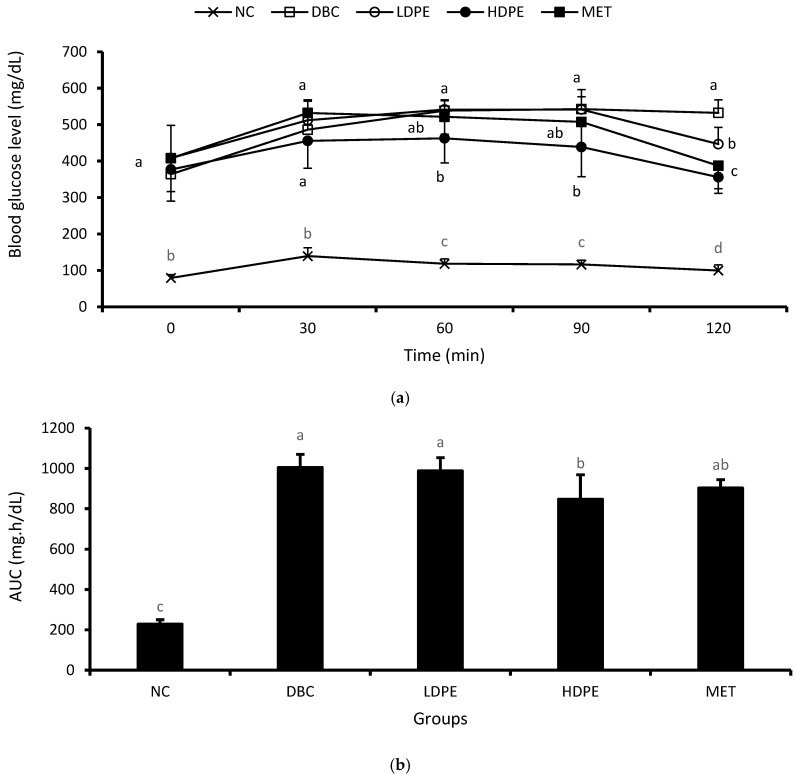
(**a**) Oral glucose tolerance data and (**b**) area under the curve. “NC”, normal control; “DBC”, diabetic control; “LDPE”, low-dose peel extract; “HDPE”, high-dose peel extract; “MET”, metformin. For each period or parameter, a statistical comparison was performed among the groups. A significant difference (*p* < 0.05) exists between any two groups when there are no similar letters.

The weekly NFBG data showed that the peel extract dose-dependently reduced NFBG over the treatment period (Figure 2). Notably, the HDPE caused a significant (*p* < 0.05) reduction in the second and fourth week of the intervention period, which was statistically comparable to that of metformin treatment, suggesting the blood glucose lowering potential of the peel’s ethyl acetate extract. Furthermore, the extract also exhibited a dose-dependent glucose tolerance effect (Figure 3a), with the effect of the HDPE being significant (*p* < 0.05) relative to the untreated diabetic animals (Figure 3b).

While the blood glucose-lowering effect of litchi peel ethyl acetate extract has been previously documented, the effect was demonstrated in alloxan-induced diabetic rats [18]. The alloxan-induced and STZ-induced diabetic models are characterized by oxidative stress-mediated pancreatic β-cell damage and impaired insulin secretion [27], which suggests that the previously documented blood glucose lowering effect of litchi peel ethyl acetate extract [18] may be influenced by an improvement in pancreatic β-cell damage, among other possible mechanisms. This is because supporting antioxidant data from the study by Chauhan et al. [18] showed that the ethyl acetate extract improved the pancreatic antioxidant status of the alloxan-induced diabetic rats.

**Figure 4:**
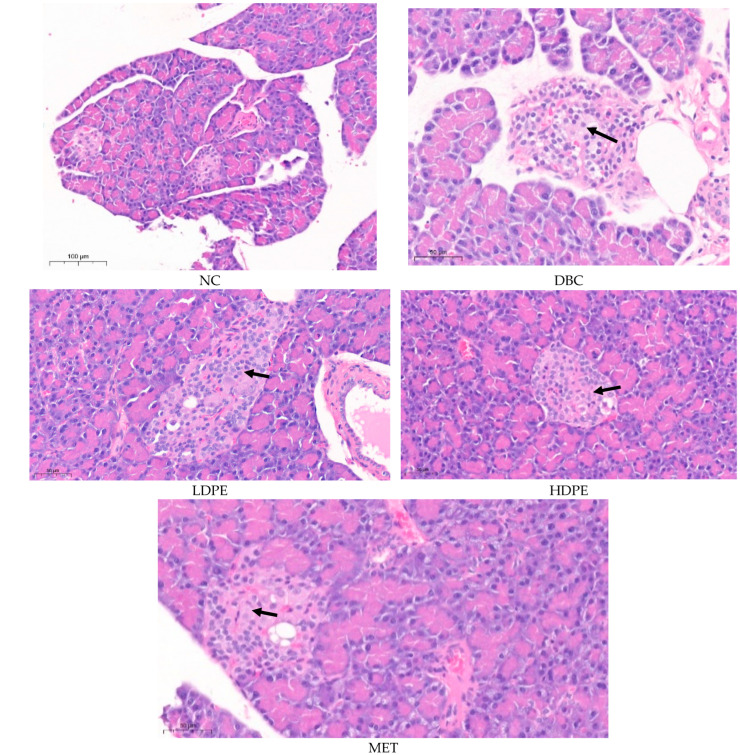
Histological images of the pancreatic tissues from rats. “NC”, normal control; “DBC”, diabetic control; “LDPE”, low-dose peel extract; “HDPE”, high-dose peel extract; “MET”, metformin.

Histology data comparing the pancreas of the normal rats (NC) with that of the untreated (DBC) and treated (LDPE, HDPE, and MET) diabetic rats further reiterate the possible ameliorative effect of litchi peel ethyl acetate extract on chemical (STZ)-induced pancreatic damage (Figure 4). The pancreatic histology of the normal rats (NC) showed that the exocrine pancreatic acinar cells appear normal and active. The available endocrine islets consisted of normal cells. The distribution pattern and size of the endocrine islets of Langerhans are considered normal with no sign of cellular or exudate accumulation (Figure 4).

The pancreatic histology of the untreated diabetic rats (DBC) showed features consistent with multifocal and mild pancreatic endocrine islet cell degeneration, which was caused by the low-dose (40 mg/kg bw) STZ treatment during induction [27]. Typically, there were mild degenerative changes consisting of cytoplasmic vacuolization with mild swelling, mild lesions, and possible atrophy within cells of the pancreatic islet (Figure 4). Similarly, the pancreatic histology of the rats treated with peel extract and metformin (MET) showed features consistent with multifocal and mild pancreatic endocrine islet cell degeneration. However, the degeneration in the pancreatic islets of rats in the HDPE group was minimal compared to that of the animals in the other groups (Figure 4), suggesting a somewhat ameliorated effect of the HDPE on STZ-induced pancreatic damage. This ameliorative effect further reflects the ability of the HDPE to significantly (*p* < 0.05) improve insulin secretion in diabetic rats as shown by the significantly (*p* < 0.05) higher serum insulin level compared to the untreated diabetic rats (Table 3).

It is plausible that the ameliorative effect of the HDPE on STZ-induced pancreatic damage may be linked to their pancreatic antioxidant capacities. The data of this study showed that the HDPE treatment significantly (*p* < 0.05) reduced pancreatic lipid peroxidation and concomitantly increased (*p* < 0.05) systemic and pancreatic catalase and SOD activities (Table 4).

Catalase and SOD are important antioxidant enzymes. SOD catalyzes the reduction of deleterious superoxide ions to hydrogen peroxide, which is reduced to water by the catalytic action of catalase [28]. Hence, the significantly higher pancreatic catalase and SOD activity of rats in the HDPE group, relative to the untreated diabetic rats, suggests that the HDPE-ameliorated STZ-induced ROS damage in the pancreatic tissue by mopping deleterious ROS. The potent in vivo antioxidant activity of the HDPE may be linked to the notable presence of bioactive flavonoids including catechins, proanthocyanidins, and procyanidins (Table 1 and Figure 1). Moreover, the hydroxyl radical and superoxide anion scavenging activity of proanthocyanidin B4, proanthocyanidin B2, and epicatechin isolated from litchi peel has been reported [11], while the modulatory effect of catechins and proanthocyanidins in vivo antioxidant status and antioxidant enzymes activity have been documented [29]. Furthermore, the key bioactive flavonoids present in the peel extracts may also influence its modulatory effect on insulin secretion. This is because a study has shown that epicatechin, a major flavonoid in the peel extract (Table 1 and Figure 1), modulates glucose-stimulated insulin secretion in INS-1 cells [30,31].

Other than the improvement in pancreatic antioxidant capacity, histology, and insulin secretion, our study further suggests that there are other possible mechanisms by which the litchi peel ethyl acetate extract improves glycemic control and ameliorates diabetic pathologies, which could be linked to the improvement in insulin action and glucose metabolism. In this study, we used fructose and low dose (40 mg/kg bw) STZ to induce diabetes, resulting in a model characterized by pancreatic β-cell damage and insulin resistance [20]. Moreover, the association between fructose consumption and insulin resistance in peripheral tissues, including the liver and muscle, has been documented [32]. Supporting data from our study showed that diabetes induction significantly (*p* < 0.05) reduced liver and muscle glycogen content (Table 3). Hepatic and muscle glycogen storage is one of the insulin-mediated mechanisms for glycemic control and glucose homeostasis [33], suggesting that diabetes induction causes impaired insulin action or signaling. However, the litchi peel extract caused a dose-dependent increase in liver and muscle glycogen content in the diabetic rats. Although the effect of the litchi peel extract was not significant (*p* ˃ 0.05), the effect of high-dose treatment (HDPE) was comparable to that of metformin treatment (Table 3), suggesting that litchi peel ethyl acetate extract potentiates glycemic control by promoting glycogen storage.

**Figure 5:**
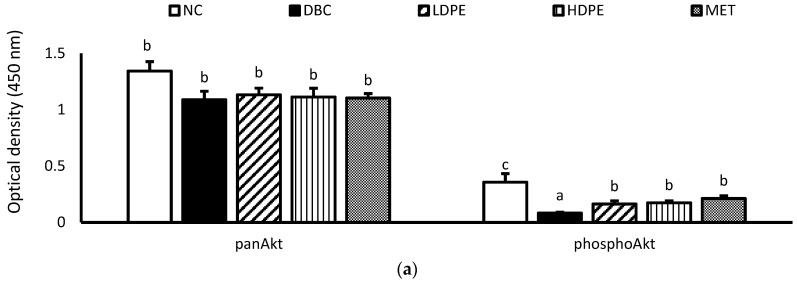

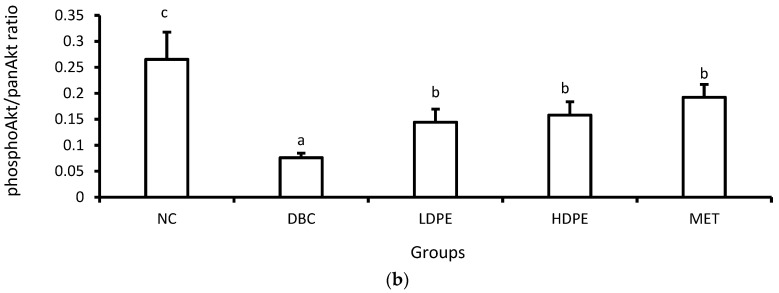
Muscle tissue (**a**) panAkt and phosphoAkt levels and (**b**) phosphoAkt/panAkt ratio of rats. “NC”, normal control; “DBC”, diabetic control; “LDPE”, low-dose peel extract; “HDPE”, high-dose peel extract; “MET”, metformin. For each parameter, statistical comparison was conducted among the groups. A significant difference (*p* < 0.05) exists between any two groups when there are no similar letters.

Furthermore, diabetes induction significantly (*p* < 0.05) reduced muscle hexokinase activity (Table 3). Hexokinase is a glycolytic enzyme that initiates the cellular utilization of glucose taken up into cells of peripheral tissues [34]. Glucose uptake and utilization are key insulin-mediated mechanisms for glucose homeostasis and glycemic control [33,34], suggesting that diabetes induction suppressed insulin-mediated glucose uptake and utilization in the muscle. Additional molecular investigation showed that diabetes induction significantly (*p* < 0.05) reduced muscle Akt phosphorylation (Figure 5), a key molecular step in the signaling of cell glucose uptake by insulin [35], buttressing the suppressive impact of diabetes induction on insulin-mediated glucose uptake and utilization in the muscle.

However, treatment with the peel extract, especially the high dose (HDPE), notably improved muscle hexokinase activity and Akt phosphorylation (Figure 5). Its modulatory effect on Akt phosphorylation was significant (*p* < 0.05) and comparable to that of metformin treatment, suggesting that the bioactive phytoconstituents in litchi peel ethyl acetate extract exerts a glycemic control effect via modulating cellular insulin signaling, glucose uptake, and utilization. Moreover, documented evidence has reported that epicatechin potentiates glycemic control by modulating the IRS-1/PI3K/Akt signal transduction pathways, thus promoting glucose transporter type 4 (GLUT-4)-mediated cellular glucose uptake [29,31].

The comparative glycemic control potential of HDPE relative to metformin may be influenced by the observed multi-mode potential of HDPE. This includes improving pancreatic histology and antioxidant status, insulin secretion and signaling, tissue glycogen production, and cellular glucose uptake and utilization. While metformin may be a known standard drug, it may not be able to ameliorate some of the STZ-induced pathologies in the diabetic animals of this study. Documented evidence has shown that metformin could improve hepatic glycogen levels by suppressing glucagon action and gluconeogenesis [36]. It could also improve insulin signaling, as well as glucose uptake and utilization, thus improving glycemic control [36]. These modes of action are consistent with the glycogen content, Akt phosphorylation, and hexokinase activity data of the present study. However, metformin is not able to affect the STZ-induced pathologies (pancreatic oxidative stress, β-cell damage, and reduced insulin production) in diabetic rats because these are outside its pharmacological mechanisms. This could be the possible reason why metformin could not normalize the blood glucose levels, or glycemic control effect of the diabetic rat model used in the present study.

## 4. Conclusions

In the present study, the antidiabetic and antioxidant potential of litchi peel ethyl acetate extract was demonstrated in a fructose-STZ model of diabetic rats. The data of the study suggests the extract potentiated glycemic control by improving pancreatic histology and antioxidant status, improving insulin secretion, promoting glycogen storage, and modulating cellular glucose uptake and utilization. LC-MS analysis further revealed that flavonoids, including catechins and proanthocyanidins, were the major phytoconstituents of the extract, which, according to documented reports, are the possible influencing bioactive components of the extract. The ethyl acetate extract of litchi peel could be a source of bioactive flavonoids that can potentiate glycemic control in diabetes and mitigate oxidative-stress-related pathologies.

## Figures and Tables

**Table 1:** LC-MS data of litchi peel ethyl acetate extract.

Identified Compounds	Ontology	ART (min)	AMz	Total Score	Peak Height	Conc. (mg/kg)
Aralidioside	Iridoid O-glycosides	5.648	447.11478	6.9378	12,228	502
Cinnamtannin B2	Flavonoids	5.923	575.11932	7.0967	17,074	701
Procyanidin B5	Flavonoids	6.635	577.13574	8.5544	6652	273
Epicatechin	Flavonoids	6.952	289.07196	8.1009	39,675	1,630
Epicatechin-(4beta->8)-epicatechin-(2beta->7,4beta->8)-catechin	Flavonoids	7.168	863.18451	7.0737	286,371	11,763
Aesculitannin G	Flavonoids	7.917	574.11267	5.7575	90,599	3721
Proanthocyanidin A2	Flavonoids	8.306	575.12164	7.9145	110,862	4554
Quercetin 3-galactoside	Flavonoid-3-O-glycosides	8.447	463.08835	6.7863	10,707	440
epi-12-Palmatoside G	Naphthopyranone glycosides	8.671	491.19287	5.8164	11,744	482
Astragalin 7-rhamnoside	Flavonoid-7-O-glycosides	8.82	593.1521	7.0845	16,335	671

“ART”, average retention time”; “AMz”, average mass-to-charge ratio; “Conc.”, concentration.

**Table 2:** Food intake, water intake, and weekly body weight.

Groups	Body Weight (g)						Food Intake (g/rat/d)	Water Intake (mL/rat/d)
	Week 0	Week 1	Week 2	Week 3	Week 3	Week 5		
NC	281 ± 9.97	307 ± 15.3 ^b^	323 ± 17.7 ^b^	324 ± 18.8 ^b^	320 ± 19.5 ^b^	328 ± 20.4 ^b^	23.0 ± 1.25 ^c^	47.7 ± 8.52 ^b^
DBC	294 ± 16.1	279 ± 24.2 ^a^	271 ± 25.9 ^a^	256 ± 23.9 ^a^	252 ± 28.0 ^a^	245 ± 20.8 ^a^	46.3 ± 2.86 ^a^	160 ± 30.8 ^a^
LDPE	291 ± 23.6	279 ± 18.4 ^a^	279 ± 26.3 ^a^	268 ± 23.5 ^a^	257 ± 25.3 ^a^	254 ± 32.0 ^a^	37.6 ± 8.13 ^b^	159 ± 26.6 ^a^
HDPE	306 ± 11.2	289 ± 18.6 ^a^	282 ± 21.9 ^a^	273 ± 28.3 ^a^	265 ± 26.5 ^a^	265 ± 28.6 ^a^	35.2 ± 1.19 ^b^	136 ± 3.61 ^a^
MET	302 ± 17.3	285 ± 20.3 ^a^	268 ± 23.6 ^a^	261 ± 24.8 ^a^	252 ± 25.8 ^a^	259 ± 29.4 ^a^	40.9 ± 5.66 ^ab^	152 ± 5.87 ^a^

“NC”, normal control; “DBC”, diabetic control; “LDPE”, low-dose peel extract; “HDPE”, high-dose peel extract; “MET”, metformin. Week 0 is the STZ injection; Week 1 is the diabetes confirmation; Week 2 to Week 5 is the intervention period. For each parameter, a statistical comparison was conducted among the groups. A significant difference (*p* < 0.05) exists between any two groups when there are no similar letters.

**Table 3:** Serum insulin, tissue glycogen content, and muscle tissue hexokinase activity.

Groups	Serum Insulin Level (µIU/mL)	Glycogen Content (mg/g tissue)		Muscle Hexokinase Activity (nmol/min/mg protein)
		Liver	Muscle	
NC	2.43 ± 0.31 ^b^	17.3 ± 3.56 ^c^	1.35 ± 0.23 ^b^	19.0 ± 5.33 ^b^
DBC	1.16 ± 0.36 ^a^	9.61 ± 1.19 ^a^	0.88 ± 0.08 ^a^	10.2 ± 1.68 ^a^
LDPE	1.50 ± 0.16 ^a^	12.4 ± 4.65 ^ab^	0.95 ± 0.19 ^ab^	11.8 ± 3.58 ^a^
HDPE	2.17 ± 0.51 ^b^	14.6 ± 2.40 ^abc^	1.10 ± 0.20 ^ab^	15.3 ± 2.00 ^ab^
MET	1.12 ± 0.11 ^a^	16.2 ± 4.68 ^c^	1.30 ± 0.15 ^b^	16.5 ± 5.07 ^ab^

“NC”, normal control; “DBC”, diabetic control; “LDPE”, low-dose peel extract; “HDPE”, high-dose peel extract; “MET”, metformin. For each parameter, a statistical comparison was conducted among the groups. A significant difference (*p* < 0.05) exists between any two groups when there are no similar letters.

**Table 4:** Lipid peroxidation and antioxidant enzyme activity in the serum and tissue of rats.

Groups	TBARS Level (µM MDS Equivalent)			SOD Activity (% Inhibition rate/mg Protein)			Catalase Activity (µmol/min/mg Protein)		
	Serum	Liver	Pancreas	Serum	Liver	Pancreas	Serum	Liver	Pancreas
NC	140 ± 28.0 ^c^	78.0 ± 16.9 ^c^	123 ± 37.3 ^c^	90.1 ± 6.90 ^b^	79.4 ± 5.91 ^b^	72.2 ± 8.19 ^c^	45.8 ± 7.24 ^b^	126 ± 13.8	47.3 ± 4.00 ^d^
DBC	564 ± 143 ^a^	234 ± 23.0 ^a^	294 ± 70.7 ^a^	66.3 ± 4.09 ^a^	66.4 ± 6.28 ^a^	44.4 ± 3.89 ^a^	27.3 ± 5.77 ^a^	112 ± 24.4	15.2 ± 4.67 ^a^
LDPE	419 ± 42.4 ^ab^	209 ± 62.4 ^ab^	222 ± 53.6 ^ab^	84.0 ± 8.71 ^b^	69.1 ± 1.24 ^a^	48.2 ± 3.38 ^a^	33.3 ± 6.67 ^a^	123 ± 15.7	26.0 ± 2.32 ^b^
HDPE	305 ± 62.1 ^a^	146 ± 22.2 ^bc^	210 ± 20.2 ^b^	85.8 ± 5.95 ^b^	74.7 ± 4.25 ^ab^	57.5 ± 2.00 ^b^	35.4 ± 3.08 ^ab^	131 ± 16.6	38.2 ± 3.85 ^c^
MET	411 ± 117 ^b^	217 ± 71.5 ^ab^	257 ± 37.9 ^ab^	70.5 ± 6.59 ^a^	68.3 ± 7.80 ^a^	47.4 ± 4.46 ^a^	30.7 ± 9.03 ^a^	116 ± 8.42	22.9 ± 8.35 ^ab^

“NC”, normal control; “DBC”, diabetic control; “LDPE”, low-dose peel extract; “HDPE”, high-dose peel extract; “MET”, metformin. For each parameter, a statistical comparison was performed among the groups. A significant difference (*p* < 0.05) exists between any two groups when there are no similar letters.

## Data Availability

The data of this study are available from the corresponding author on request.
